# Oligodendroglia in cortical multiple sclerosis lesions decrease with disease progression, but regenerate after repeated experimental demyelination

**DOI:** 10.1007/s00401-014-1260-8

**Published:** 2014-02-25

**Authors:** Enrique Garea Rodriguez, Christiane Wegner, Mario Kreutzfeldt, Katharina Neid, Dietmar R. Thal, Tanja Jürgens, Wolfgang Brück, Christine Stadelmann, Doron Merkler

**Affiliations:** 1 Institute of Neuropathology, University Medical Center Göttingen, Robert-Koch-Str. 40, 37099 Göttingen, Germany; 2 Division of Clinical Pathology, Geneva University Hospital, Centre Médical Universitaire, 1, rue Michel Servet, 1211 Geneva, Switzerland; 3 Department of Pathology and Immunology, Geneva University, Geneva, Switzerland; 4 Institute of Pathology/Laboratory for Neuropathology, University of Ulm, Ulm, Germany; 5 Present Address: Department of Neuroanatomy, Institute of Anatomy and Cell Biology, University of Freiburg, Freiburg, Germany

**Keywords:** Multiple sclerosis, Cortical demyelination, Oligodendrocytes, Oligodendrocyte precursors, Targeted cortical EAE

## Abstract

Cerebral cortex shows a high endogenous propensity for remyelination. Yet, widespread subpial cortical demyelination (SCD) is a common feature in progressive multiple sclerosis (MS) and can already be found in early MS. In the present study, we compared oligodendroglial loss in SCD in early and chronic MS. Furthermore, we addressed in an experimental model whether repeated episodes of inflammatory SCD could alter oligodendroglial repopulation and subsequently lead to persistently demyelinated cortical lesions. NogoA^+^ mature oligodendrocytes and Olig2^+^ oligodendrocyte precursor cells were examined in SCD in patients with early and chronic MS, normal-appearing MS cortex, and control cortex as well as in the rat model of repeated targeted cortical experimental autoimmune encephalomyelitis (EAE). NogoA^+^ and Olig2^+^ cells were significantly reduced in SCD in patients with chronic, but not early MS. Repeated induction of SCD in rats resulted only in a transient loss of NogoA^+^, but not Olig2^+^ cells during the demyelination phase. This phase was followed by complete oligodendroglial repopulation and remyelination, even after four episodes of demyelination. Our data indicate efficient oligodendroglial repopulation in subpial cortical lesions in rats after repeated SCD that was similar to early, but not chronic MS cases. Accordingly, four cycles of experimental de- and remyelination were not sufficient to induce sustained remyelination failure as found in chronic cortical MS lesions. This suggests that alternative mechanisms contribute to oligodendrocyte depletion in chronic cortical demyelination in MS.

**Electronic supplementary material:**

The online version of this article (doi:10.1007/s00401-014-1260-8) contains supplementary material, which is available to authorized users.

## Introduction

Multiple sclerosis (MS) is characterized by multifocal inflammatory demyelinating lesions in white and gray matter of the central nervous system (CNS) [31]. Neocortical demyelination in particular is a frequent phenomenon found in 90 % of patients with chronic MS [30, 50], but can already be present in patients with early MS [34]. Despite early description of neocortical lesions in MS [6], these lesions have mostly been overlooked until recently since they are difficult to detect with traditional staining techniques [41] or conventional magnetic resonance imaging (MRI) [17, 27]. The use of new MRI sequences has shown that cortical lesions in MS are more common than previously thought [8, 17, 26] and are supposed to contribute to cognitive impairment [7, 44] and epilepsy [9] in MS. Therefore, cortical involvement may represent an important pathophysiological substrate for disease manifestation, progression and therapy in MS [12, 18, 30].

On average, between 10 and 25 % of cortical gray matter (GM) is demyelinated in patients with chronic MS, but individual patients may even reach values of over 70 % [5, 19, 30]. During the progressive stage of the disease, the extent of GM demyelination can even exceed that of white matter (WM) demyelination [19, 30]. Histopathologically, three main types of cortical lesions have been described according to their topographic distribution within the cerebral cortex: leukocortical (type I) lesions extending through WM and GM, purely intracortical (type II) lesions and subpial (type III) lesions [41]. Subpial cortical demyelination (SCD), the most frequent lesion type in chronic MS, accounts for the majority of demyelinated cortex and seems to occur independent of WM demyelination [3, 30]. Compared to WM lesions, cortical demyelination presents with less lymphocyte infiltration and reduced microglia activation [4, 41, 48]. However, the extent of meningeal inflammation was shown to correlate with the extent of cortical demyelination and rate of progression in patients with secondary progressive MS [24, 35, 36]. Similarly, meningeal and intracortical infiltrates were topographically associated with cortical lesions in early-stage MS, which supports the concept that inflammatory processes underlie cortical demyelination [34].

Despite the observation that GM lesions have a higher remyelination propensity than WM lesions [2, 12], chronic cortical demyelinated lesions accumulate over time and are most widespread in progressive MS [30]. It has been postulated that the depletion of oligodendrocyte precursor cells (OPCs) [10] and oligodendroglial differentiation block [15, 29] could account for remyelination failure in WM lesions. Previous experimental studies furthermore suggested that repeated rounds of de- and remyelination lead to less efficient remyelination in WM [25].

In the present study, we analyzed cortical oligodendrocytes (OLs) and OPCs in early- and late-stage MS. Our findings reveal that both mature OLs and their precursors are significantly reduced in demyelinated cerebral cortex in late-stage, but not in early MS. To address whether repeated cycles of cortical de- and remyelination could account for the depletion of OLs and lack of remyelination, we used a rat model of targeted cortical experimental autoimmune encephalomyelitis (EAE) which recreates key histopathological features of cortical MS lesions [38]. Here, we induced up to four consecutive cortical EAE lesions within the same cortical area and investigated the impact on repopulation of OLs and remyelination. We found that the differentiation of OPCs into mature OLs was not sustainably impaired even after repeated cortical de- and remyelination. Our study thus suggests that cortical remyelination failure in MS is not necessarily due to repeated waves of de- and remyelination. Further studies are required to unravel the mechanisms underlying cortical remyelination failure in progressive MS.

## Materials and methods

### Clinical data and human tissue

Brain samples were fixed in 4 % formaldehyde and paraffin embedded. Blocks consisted of five autopsy cases with chronic MS [mean age 54.2 (range 41–66) years; mean disease duration 16.8 (range 10–25) years] as well as four biopsy cases with early MS [mean age 36.8 (range 26–45) years; mean disease duration 3.3 (range 2–6) months]. Tissue blocks from age- and sex-matched control autopsy cases without neurological disease [*n* = 10; 5 males, 5 females; mean age 50.3 (range 35–75) years] served as controls. For the detection of SCD, paraffin sections were immunostained for myelin basic protein (MBP). Clinical data are summarized in Table 1 (autopsy cases) and Table 2 (biopsy cases). All MS autopsy cases showed a progressive disease course with disease durations of at least 10 years, whereas biopsy cases suffered from early MS. Based on the number of relapses, biopsy cases were either classified as clinically isolated syndrome (CIS) (*n* = 3) or relapsing–remitting MS (*n* = 1) as indicated in Table 2. Biopsies were performed for diagnostic purposes to exclude neoplastic or infectious diseases. The study was approved by the ethics committee of the University Medical Center Göttingen (WF-004/11).

**Table 1:** Clinical findings of MS autopsy cases

MS autopsy case	Age	Sex	Disease duration (years)	Disease course
1	47	F	25	Secondary progressive
2	66	M	12	Primary progressive
3	41	M	10	Progressive
4	60	F	25	Secondary progressive
5	57	M	12	Secondary progressive

**Table 2:** Clinical findings of MS biopsy cases

MS biopsy case	Age/Sex	Disease duration (months)	Disease course	Presenting symptom(s)	Brain MRI
1	42/F	2	CIS	Vision loss, disturbed balance, depression, beginning dementia	Multiple intracerebral WM lesions, partly contrast enhancing in periventricular and subcortical areas
2	26/F	2	CIS	Minor weakness of the left hand, focal motor seizures	Several intracerebral WM lesions: three focal subcortical lesions measuring up to 3 cm in diameter, partly ring enhancing; two brain stem lesions
3	45/F	6	CIS	Slight apraxia and unsteady gait	Multiple intracerebral WM lesions in the subcortical and periventricular areas
4	34/F	3	RRMS	Blurred vision of the left eye	Multiple intracerebral WM lesions, partly contrast enhancing in the juxtacortical areas and brain stem

Based on the number of relapses, biopsy cases were either classified as clinically isolated syndrome (CIS) (patients 1–3) or relapsing–remitting MS (RRMS) (patient 4) as indicated. CIS patients underwent biopsy after the first relapse, whereas the patient with RRMS was biopsied after the second relapse

### Histology and immunohistochemistry on human tissue

Bielschowsky silver staining and immunohistochemistry were carried out according to standard procedures. The following primary antibodies were used: rabbit anti-MBP (1:1,000, Dako, Denmark), mouse anti-MBP peptide_70–89_ (SMI94; 1:3,000, Covance, Princeton, New Jersey, USA), mouse anti-KiM1P (1:5,000) [43], rabbit anti-Olig2 (1:100, IBL, Spring Lake Park, Minnesota, USA), mouse anti-NogoA (1:10,000, mAb 11C7, a generous gift from M.E. Schwab, Brain Research Institute, ETH and University of Zurich, Switzerland) and mouse anti-Ki67 (1:50, clone Mib-1, Dako, Denmark). To identify Olig2^+^ or NogoA^+^ cells in control non-MS, normal-appearing MS and demyelinated MS neocortex, we performed double-immunolabeling combining either rabbit anti-Olig2 with mouse anti-MBP_70–89_ (SMI94) or mouse anti-NogoA with rabbit anti-MBP.

Double-labeling immunohistochemistry was performed combining DAB (first primary antibody) and Fast Blue (second primary antibody with APAAP; Dako). For fluorescence double-labeling, Cy3- or Cy2-conjugated goat anti-mouse IgG and goat anti-rabbit IgG (both from Jackson ImmunoResearch Europe Ltd.) were used.

### Morphometry and statistics on human tissue

The density of KiM1P^+^, NogoA^+^ and Olig2 strongly positive (Olig2^+^) cells was determined as ≥20,000 μm^2^. KiM1P^+^ mononuclear cells of both macrophage and microglia phenotype were counted in SCD in layers I/II and in layer III and given as cells/mm^2^. The mean number of Olig2^+^ and NogoA^+^ cells was quantified in layers I/II and in layer III in control non-MS neocortex, normal-appearing MS neocortex (NAGM) as well as chronic and early SCD.

The density of axons (layer III) was assessed on Bielschowsky silver-stained sections (1000×) in control non-MS neocortex, MS NAGM as well as chronic SCD within at least four visual fields using a 10 × 10 counting grid. Axonal density was given in percent of control non-MS neocortex as a reference (set to 100 %).

### Animals and groups

A total number of 101 adult female Lewis rats (195 g ± 15, Harlan, Horst, Netherlands) were included in this study. The animals were kept in groups (maximum 8 animals per cage) on a 12:12 h light/dark cycle with food and water provided ad libitum. All experiments were approved by the Bezirksregierung Braunschweig, Germany.

### Sensitization procedure

Recombinant rat myelin oligodendrocyte glycoprotein (rMOG) was produced as described previously [1]. For subclinical immunization, anaesthetized rats (*n* = 68) were injected subcutaneously (s.c.) with rMOG (50 μg) emulsified in incomplete Freund’s adjuvant (IFA; Sigma-Aldrich Chemie GmbH, Steinheim, Germany) as described previously [38]. For control experiments, rats (*n* = 27) were injected s.c. with PBS emulsified in IFA. A subset of animals (*n* = 6) received no injection (naive controls).

### Intracerebral stereotactic injection

Stereotactic injections were performed according to a modified protocol as described previously [38]. Briefly, anaesthetized IFA- or rMOG-sensitized rats received a first stereotactic injection into the cortex (1 mm caudal to the bregma, 2 mm lateral to the sagittal suture, 1.7 mm depth) 19–21 days after immunization. 1 μl of a cytokine mixture [250 ng of recombinant rat tumor necrosis factor-α (TNF-α; R&D Systems, Abingdon, UK; 150 U of recombinant rat interferon-γ (IFN-γ; PeproTech, London, UK)] dissolved in PBS was injected together with a trace of Monastral blue (Fluka, Germany). Stereotactic cytokine injection at the same anatomical location was repeated up to four times at intervals of 21 days.

### 5-Bromo-2-deoxyuridine (BrdU) injection

BrdU labeling was performed in a subset of IFA- or rMOG-sensitized animals (*n* = 6 per group). Animals were injected twice daily intraperitoneally with 1.5 ml 0.9 % NaCl solution containing 100 mg/kg bodyweight BrdU (Sigma) for 5 days starting on day 2 after cytokine injection. At 21 days after cytokine injection, the animals were killed for histology.

### Histopathology and immunohistochemistry of rat tissue

Animals were anaesthetized by injecting an overdose of 14 % chloral hydrate (Merck). After transcardial perfusion with 4 % paraformaldehyde, brains were dissected and paraffin embedded. Serial brain sections (2 μm) were collected from the injection site. Immunohistochemistry was performed as described above. Additional antibodies used were: mouse anti-proteolipid protein (PLP, clone Plpc1, 1:2,500, Biozol), mouse anti-BrdU (1:400 in 10 % FCS, Chemicon) and mouse anti-ED1 (activated macrophages/microglia, 1:1,000, Serotec).

### Morphometric analysis of focal cortical EAE lesions

Images were captured using light and fluorescence microscopes equipped with a digital camera (Color View II and DP71, Olympus, Germany). NogoA^+^ and Olig2^+^ cell populations were determined on NogoA/MBP and Olig2/MBP double-stained sections. The density of both cell populations was assessed in cortical layers I and II (400×) using a 10 × 10 counting grid and expressed as cells per mm^2^. The size of the demyelinated area was quantified on MBP-immunostained sections using Analysis^®^ software (Analysis, Soft Imaging System, Germany).

Axonal density was quantified on Bielschowsky silver-impregnated sections as described above and given in percent of untreated controls. The fraction of myelinated axons was determined in cortical layer III within four visual fields (1000×) using a counting grid (10 × 10 squares). The numbers of MBP^+^ myelin sheaths and Bielschowsky-impregnated axons intersecting with the crosses of the counting grid were determined. The ratio of MBP^+^ fibers and Bielschowsky-impregnated axons was calculated (expressed in % of axons).

To quantify proliferated OPCs, Olig2 and BrdU double-positive cells were assessed on fluorescence images (200×) of cortical layers I/II (3 optical fields per animal). The quantification applied a custom-made script based on Cognition Network Language (Definiens Cognition Network Technology; Definiens Developer XD software) and resembled previously described scripts [28, 37]. Briefly, cells showing (a) an overlapping signal in the BrdU and Olig2 channel and (b) a signal for DAPI staining (nuclei) were counted and expressed as cells per mm^2^.

### Statistical analysis

Statistical analysis was performed using SPSS (Version 12, SPSS Inc., Chicago, IL, USA) or GraphPad Prism (GraphPad Prism Software, Ink, San Diego, California, USA). Normality of distribution was tested by Kolmogorov–Smirnov tests. Statistical calculations included one-way analysis of variance (ANOVA) if three or more groups were compared, followed by post hoc least significance difference (LSD) test as indicated in the figure legends. For comparisons between two groups, unpaired *t* tests were performed.

The subanalysis within MS autopsy cases used paired *t* tests to compare the cell and axonal densities in demyelinated MS neocortical layers versus adjacent MS NAGM. To test for altered cell densities in SCD between chronic (autopsies) and early (biopsies) MS cases, Mann–Whitney tests were performed. A probability value of less than 0.05 was considered significant. All data are expressed as mean ± standard error of the mean (SEM) and shown in graphs as mean + SEM.

## Results

### Subpial cortical demyelination in early and chronic MS

**Fig. 1:**
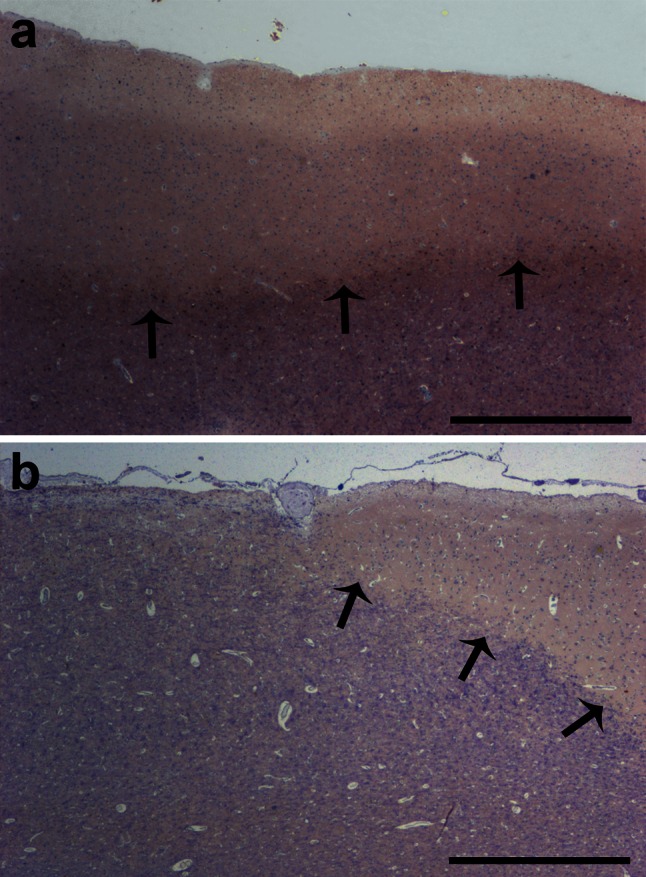
Subpial cortical demyelination in early and chronic MS cases. Immunohistochemistry for MBP (*blue*) reveals subpial cortical demyelination (SCD) in early (biopsy) (**a**) and chronic (autopsy) (**b**) cases with MS. **a** Exemplary biopsy tissue from one of the patients with early MS shows confluent SCD affecting cortical layers I–III. **b** Exemplary section from one of the autopsy cases with long-standing MS displays the transition zone of normal-appearing myelinated cortex (on the left) and SCD (on the right). *Arrows* indicate the border of the cortical demyelination. *Scale bars* 1 mm in **a** and **b**

SCD is prominent in most patients with chronic MS [30], but can already be present in early MS [34]. We detected SCD affecting layers I–III in all selected MS biopsy (*n* = 4; Fig. 1a) and autopsy (*n* = 5, Fig. 1b) cases. In addition, all MS autopsy cases also showed adjacent normal-appearing myelinated cortical layers I–III (Fig. 1b). In contrast, most tissue blocks from the investigated MS biopsy cases exhibited only demyelinated layers I–III, thereby restricting the analysis of biopsies to demyelinated areas only. All control cases displayed intact neocortical myelin.

**Fig. 2:**
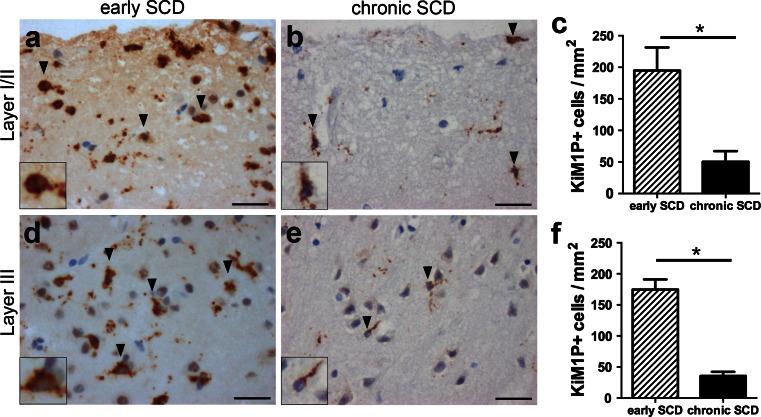
Marked microglia/macrophage activation in early, but not chronic subpial MS lesions. **a**–**f** Immunohistochemistry for KiM1P^+^ macrophages and microglia in SCD of chronic (autopsy) and early (biopsy) cases with MS. Representative images show KiM1P^+^ cells within early (**a**) and chronic (**b**) SCD of cortical layers I/II as well as the quantification thereof (**c**). Representative images show KiM1P^+^ cells in early (**d**) and chronic (**e**) SCD within cortical layer III as well as the quantification thereof (**f**). *Black arrowheads* point toward exemplary KiM1P+ cells. *Insets* in **a, b, d** and **e** show KiM1P^+^ cells in greater detail. *Scale bars* 25 μm in **a, b, d, e**; *error bars* indicate SEM. *p < 0.05

Subpial cortical lesions were classified as inactive demyelinated lesions in all MS autopsy (*n* = 5) and biopsy (*n* = 4) cases based on the absence of myelin-laden phagocytes. Since the short disease duration in biopsy cases ranged from 2 to 6 months, we investigated whether these early (biopsy) cases displayed increased macrophage and microglia infiltration in SCD compared to late (autopsy) cases. The quantitative analysis in demyelinated cortical layers I/II showed significantly higher densities of KiM1P^+^ cells in early biopsy cases (194.8 ± 36.6 cells/mm^2^) (Fig. 2a) compared to late autopsy cases with MS (50.4 ± 16.9 cells/mm^2^; *p* < 0.05) (Fig. 2b, c). Similarly, the densities of KiM1P^+^ cells in demyelinated cortical layer III were also significantly increased in early MS (biopsy) (174.6 ± 16.4 cells/mm^2^) (Fig. 2d) compared to late MS (autopsy) (35.6 ± 6.7 cells/mm^2^; *p* < 0.05) (Fig 2e, f) cases. Thus, the more pronounced microglia and macrophage activation supported the assumption that SCD noted in biopsies evolved more recently than SCD observed in autopsy brain samples.

### Marked reduction of oligodendrocytes in chronic, but not early subpial MS lesions

To investigate whether oligodendroglial densities in SCD are affected by disease duration, we quantified the density of NogoA^+^ OLs in SCD in patients with a short (early MS) or a long (chronic MS) disease duration.

**Fig. 3:**
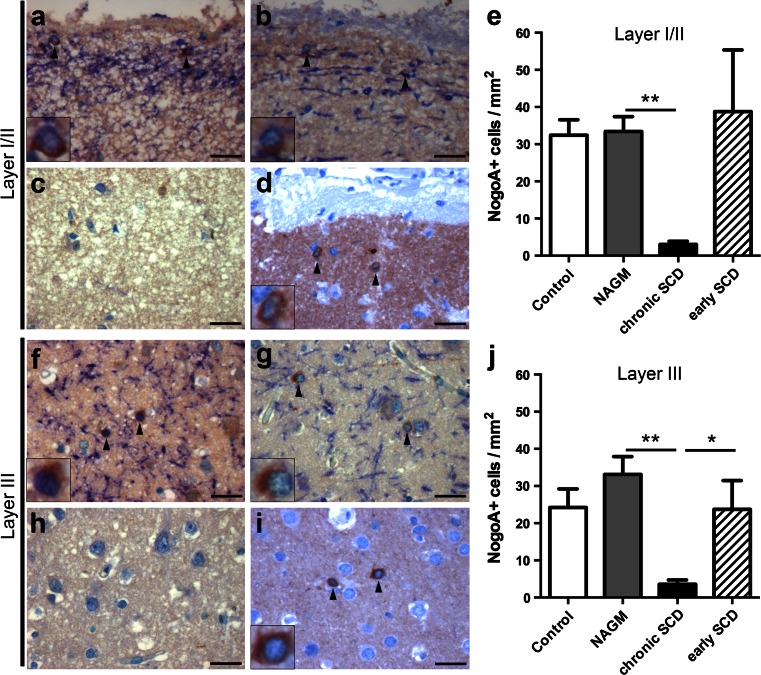
Reduction of oligodendrocytes in cortical layers I/II and layer III in chronic, but not early MS lesions. **a**–**j** Immunohistochemistry for MBP (*blue*) and NogoA^+^ OLs (*brown*) within layers I/II (**a**–**d**) or layer III (**f**–**i**), respectively, and the corresponding quantitative evaluation (**e**, **j**). Representative images of control cortex (**a**, **f**), normal-appearing cortex (NAGM) of chronic MS (**b**, **g**) as well as chronic (**c**, **h**) or early (**d**, **i**) SCD. Similar numbers of NogoA^+^ cells are noted in cortical layers I/II (**a**, **b**, **d**, **e**) and layer III (**f**, **g**, **i**, **j**) in the control cortex (**a**, **f**), NAGM (**b**, **g**) and SCD from patients with early MS (**d**, **i**). In patients with chronic MS, NogoA^+^ cells are significantly reduced in chronic SCD (**c**, **h**) in cortical layers I/II (**c**) and III (**h**) each compared to the corresponding NAGM area. The densities of NogoA^+^ OLs in layer III are significantly decreased in chronic SCD compared to early SCD (**j**), whereas there is a trend for the group comparison within layers I/II (**e**) (*p* = 0.06). NogoA^+^ cells are indicated by *black arrowheads* and shown in detail in *insets*. *Scale bars* 25 μm in **a**–**d** and **f**–**i**; *error bars* indicate SEM. *p < 0.05, **p < 0.01

We first assessed the density of NogoA^+^ OLs in the superficial layers I/II. Autopsy cases displayed similar numbers of NogoA^+^ cells in the control cortex (32.4 ± 4.2 cells/mm^2^, Fig. 3a) compared to normal-appearing MS cortex (NAGM) (33.4 ± 4 cells/mm^2^, Fig. 3b, e). SCD areas of progressive MS patients showed a significant decrease of NogoA^+^ OLs in the superficial layers (3 + 0.8 cells/mm^2^, Fig. 3c) compared to adjacent normal-appearing layers I/II (*p* < 0.01, Fig. 3b, e). In contrast, NogoA^+^ cell numbers in layers I/II were unaltered in SCD of patients with early MS (38.8 + 16.6 cells/mm^2^, Fig. 3d) as compared to control and MS NAGM (Fig. 3e). The analysis of demyelinated layers I/II showed a trend toward higher numbers of NogoA^+^ cells in patients with early versus late MS (*p* = 0.06).

We further quantified NogoA^+^ OLs in layer III in MS and control autopsy cases. MS NAGM displayed similar densities of NogoA^+^ cells (33.1 ± 4.8 cells/mm^2^) as control cortex (24.2 ± 5 cells/mm^2^) in layer III (Fig. 3f, g). In progressive MS patients, NogoA^+^ OLs were significantly reduced in demyelinated layer III (3.6 ± 1.1 cells/mm^2^, Fig. 3h) compared to adjacent normal-appearing layer III (*p* < 0.01, Fig. 3g, j). In contrast, patients with early MS showed similar oligodendroglial numbers in demyelinated layer III (23.7 ± 7.8 cells/mm^2^, Fig. 3i) as control and MS NAGM (Fig. 3j). Significantly more NogoA^+^ OLs were detected in demyelinated layer III in patients with early disease than in patients with chronic MS (*p* < 0.05).

### Reduced density of oligodendrocyte precursor cells (OPCs) in chronic, but not early MS lesions

To address whether the density of OPCs in subpial cortical lesions is affected by disease duration, we quantified OPCs in SCD in early versus late MS. Olig2 is strongly expressed in OPCs and weakly in mature OLs in the adult human CNS [29]. Thus, we only counted the number of Olig2 strong^+^ cells (referred to as Olig2^+^ cells).

**Fig. 4:**
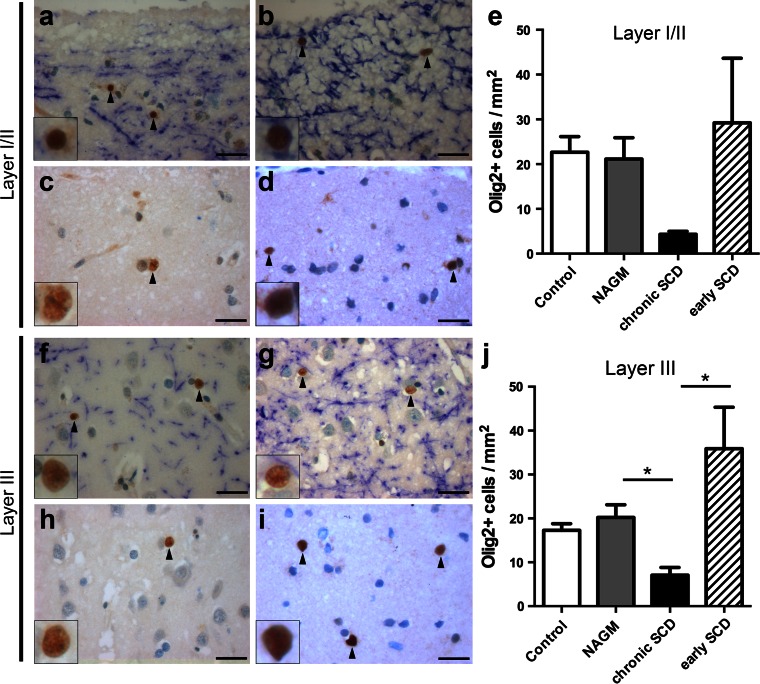
Reduced oligodendrocyte precursors in chronic, but not early MS lesions. **a**–**j** Immunohistochemistry for OPCs with strong Olig2 expression (Olig2^+^ cells) (*brown*) and MBP-positive myelin (*blue*) within layers I/II (**a**–**d**) or layer III (**f**–**i**), respectively, and the corresponding quantitative evaluation (**e**, **j**). Representative images are shown of control cortex (**a**, **f**), normal-appearing cortex (NAGM) of chronic MS (**b**, **g**) as well as chronic (**c**, **h**) and early (**d**, **i**) SCD. Similar numbers of Olig2^+^ cells are noted in myelinated cortical layers I/II (**e**) or layer III (**j**) in control cortex, MS NAGM and SCD from patients with early MS. In layers I/II, there is a trend for decreased Olig2^+^ OPCs in chronic SCD (autopsy) (**c**) compared to either adjacent NAGM (*p* = 0.05) or demyelinated early SCD (biopsy) (*p* = 0.06) (**e**). In layer III Olig2^+^ cells are significantly reduced in chronic SCD (**h**, **j**) compared to NAGM and early SCD (*p* < 0.05 for both comparisons). Olig2^+^ cells are indicated by *black arrowheads* and shown in detail in *insets*. *Scale bars* 25 μm in **a**–**d** and **f**–**i**; *error bars* indicate SEM. *p < 0.05

The densities of Olig2^+^ cells in the superficial layers I/II were similar in control (22.7 ± 3.5 cells/mm^2^, Fig. 4a) and MS NAGM (21.1 ± 4.8 cells/mm^2^, Fig. 4b, e). Within progressive MS cases, Olig2^+^ cell numbers were decreased in demyelinated layers I/II (4.3 ± 0.7 cells/mm^2^, Fig. 4c) compared to adjacent normal-appearing layers I/II (*p* = 0.05, Fig. 4b, e). In contrast, OPC density was not altered in early SCD of patients with early MS (29.3 ± 14.4 cells/mm^2^, Fig. 4d) and showed a trend toward higher densities compared to chronic SCD (*p* = 0.06, Fig. 4e). However, we could not detect Olig2^+^ cells that co-expressed the proliferation marker Ki67 within SCD in these biopsies, indicating that OPCs might have proliferated already before the bioptic surgery took place (data not shown).

We also assessed Olig2^+^ progenitors in the outer pyramidal layer III. The control (17.3 ± 1.5 cells/mm^2^, Fig. 4f) and MS NAGM (20.2 ± 2.9 cells/mm^2^, Fig. 4g) displayed similar densities of Olig2^+^ OPCs in layer III. In chronic MS cases, there was a significant reduction of Olig2^+^ cells in demyelinated layer III (7.1 ± 1.8 cells/mm^2^, Fig. 4h) compared to adjacent normal-appearing layer III (*p* < 0.05, Fig. 4g, j). In contrast, early SCD showed no reduction of Olig2^+^ cells in layer III (35.9 ± 9.4 cells/mm^2^, Fig. 4i) compared to control (Fig. 4f) and MS NAGM (Fig. 4g, j). The density of Olig2^+^ cells in layer III was significantly reduced in chronic compared to early SCD (*p* < 0.05, Fig. 4j). Furthermore, the quantification of axonal densities in layer III of chronic SCD did not reveal any significant reduction compared to controls or NAGM (Suppl. Fig. 1). Taken together, these results suggest that OLs and their precursors are not reduced in SCD in early MS, but show a marked decline in progressive disease.

### Efficient oligodendroglial regeneration after recurrent demyelinating episodes in rats with targeted cortical EAE lesions

**Fig. 5:**
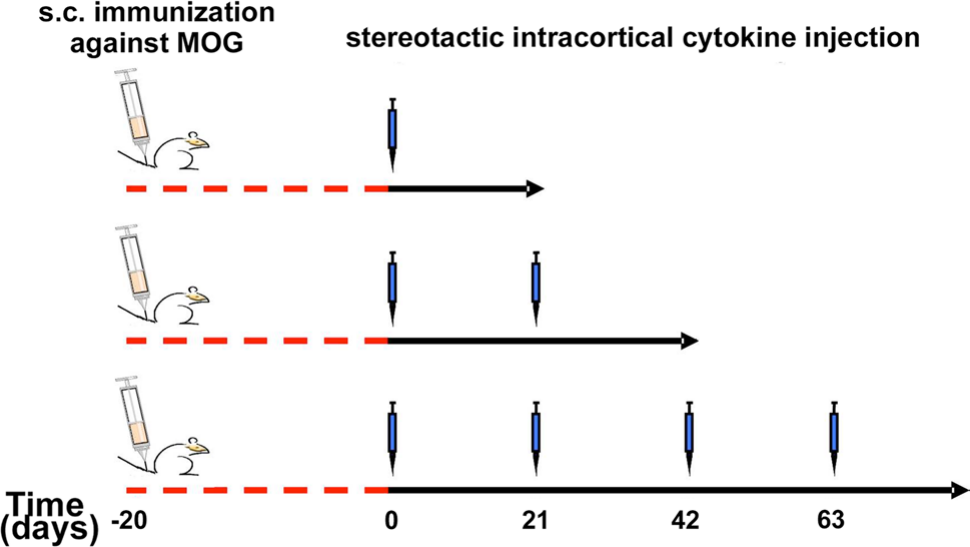
Experimental setup for cortical targeted EAE induction in rats. Animals were immunized with rMOG emulsified in incomplete Freund’s adjuvant (IFA). Controls (not depicted in the graph) were immunized with IFA only. 20 days later, cytokines were stereotactically injected into the cortex either once (*top row*), twice (*middle row*) or four times (*bottom row*) with 3 weeks time interval(s) between each injection

**Fig. 6:**
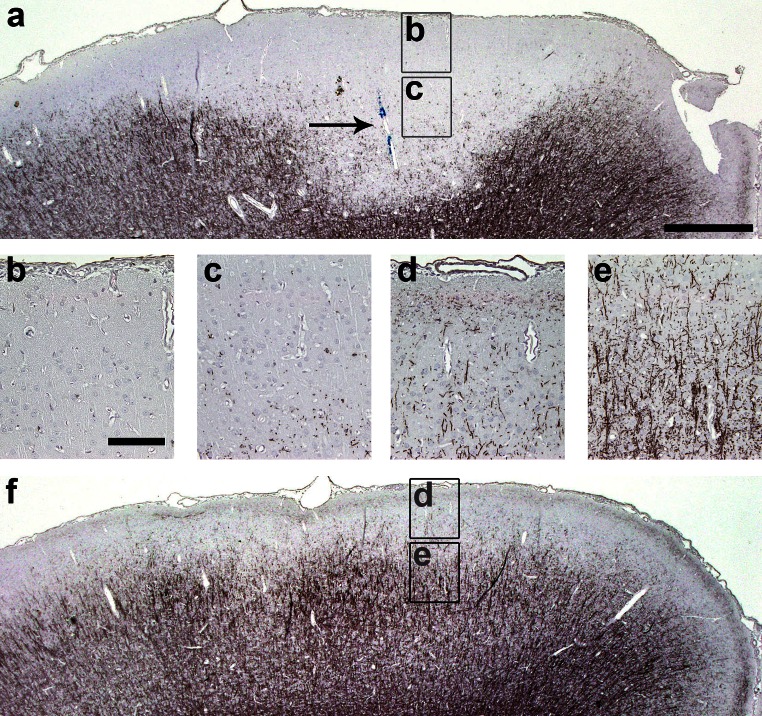
Focal subpial cortical demyelination in the ipsilateral, but not contralateral cortex after intracerebral cytokine injection in rMOG-immunized rats. **a**–**f** Representative photographs of MBP-immunostained cortex of rMOG-primed rats on day 3 post-lesion induction (**a**–**c**). Extensive ipsilateral demyelination is present 3 days after intracortical cytokine injection in rMOG-immunized rats. Targeted lesion is indicated by *arrow* marking the injected *blue dye*. **d**–**f** In contrast, the contralateral non-injected hemisphere displays intact myelin. *Scale bars* **a** and **f** 500 μm, **b**–**e** 100 μm

We aimed to examine experimentally whether consecutive episodes of SCD might lead to loss of oligodendroglial cells and exhaustion of remyelination. Therefore, we induced repeated SCD within the same anatomical area in an established animal model of MS. In this model, extensive cortical remyelination has already been demonstrated after a single demyelinating episode [38]. In the present study, we induced up to four consecutive targeted cortical EAE lesions by repeated stereotactic intracortical cytokine injection (for experimental setup see Fig. 5). As described previously [38], rMOG-immunized rats showed extensive SCD on day 3 after a single intracerebral cytokine injection (Fig. 6a–c). SCD extended along the pial surface of the ipsilateral hemisphere, reminiscent of subpial type III cortical MS lesions. In contrast, the contralateral cortex was devoid of demyelination (Fig. 6d–f).

**Fig. 7:**
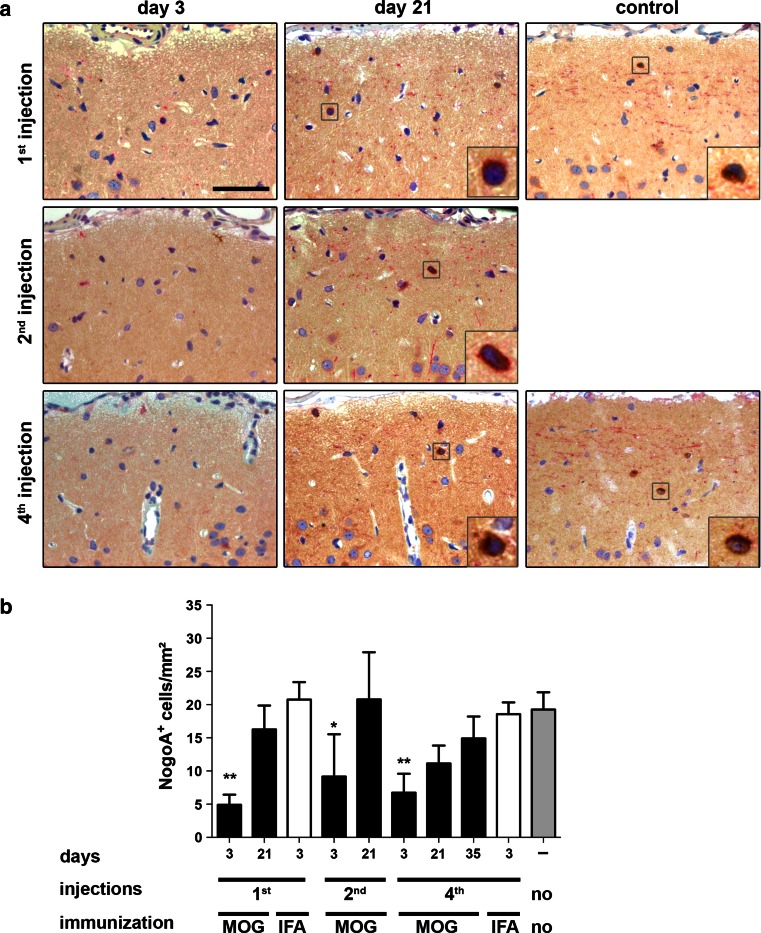
Efficient repopulation with cortical NogoA^+^ oligodendrocytes after repeated cycles of cortical demyelination in rats. **a** Representative photographs of NogoA-immunostained sections of lesioned subpial cortex at indicated time points and in different animal groups. **b** Quantification of NogoA^+^ cells. At 3 days post-lesion induction, NogoA^+^ cell density is significantly decreased followed by significant repopulation (on day 21) after one and two demyelinating events. After four episodes of cortical lesion induction, a trend toward lower OLs counts (*p* = 0.052) is noted in comparison to naive controls. However, 35 days after the fourth lesion induction the density increases again to control levels. Data are expressed as mean + SEM. For statistical evaluation, one-way ANOVA followed by post hoc LSD test is performed (*p < 0.05, **p < 0.01). *Scale bar* 50 μm, length of enlarged image 14 μm

To assess the impact of repetitive SCD on oligodendroglial regeneration, we then performed repeated intracortical cytokine injections every 3 weeks up to four times into the same anatomical area (see experimental setup Fig. 5). We first assessed the densities of NogoA^+^ OLs at the different experimental time points and conditions. Three days after the last cytokine injection, NogoA^+^ OLs were significantly reduced in rMOG-immunized rats with one (4.9 ± 1.5 cells/mm^2^; *p* < 0.01), two (9.2 ± 2.8 cells/mm^2^, *p* < 0.05) and four (6.8 ± 2.8 cells/mm^2^; *p* < 0.01) demyelinating episodes compared to untreated controls (19.3 ± 2.1 cells/mm^2^) (Fig. 7a, b). After this initial drop of OLs, a substantial repopulation of NogoA^+^ cells was observed in rMOG-immunized animals 3 weeks after single, twofold and fourfold lesion induction (Fig. 7b). Three weeks after the last injection, animals with one (16.3 ± 3.6 cells/mm^2^) and two (20.8 ± 3.5 cells/mm^2^) demyelinating episodes showed similar densities of NogoA^+^ cells as untreated controls. Three weeks following the fourth lesion, there was a trend toward reduced NogoA^+^ cells compared to age-matched controls (11.1 ± 2.7 cells/mm^2^ versus 19.3 ± 2.6 cells/mm^2^; *p* = 0.052). To investigate whether this trend reflected a final stage or rather a delay in oligodendroglial recruitment, NogoA^+^ cells were quantified later at 35 days after the fourth injection in rMOG-immunized rats (Fig. 7b). At this later time point, NogoA^+^ cell density was similar to control levels (*p* = 0.36) (Fig. 7b).

**Fig. 8:**
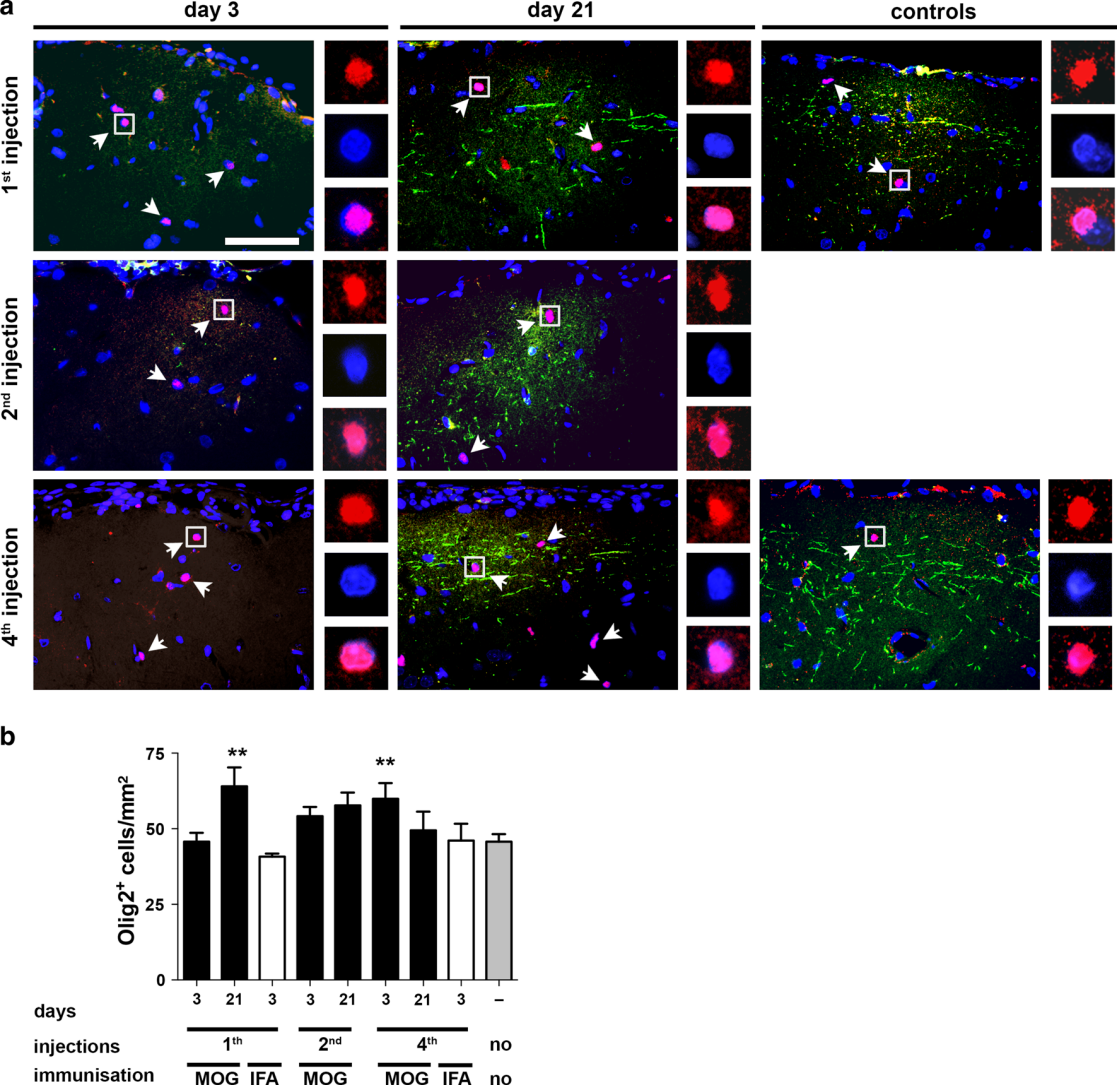
No reduction of Olig2^+^ oligodendrocyte precursor population after repeated demyelination in rats. **a** Representative photographs of Olig2 and PLP double-immunostained sections within the cytokine-injected subpial cortex. Merged overview photographs show Olig2^+^ precursors (*red*), PLP^+^ myelin (*green*) and DAPI^+^ nuclei (*blue*). *Arrows* point toward Olig2^+^ OPCs and *white squares* indicate the area from which the insets are taken (upper image Olig2, middle: DAPI, lower merged image). **b** No significant reduction of Olig2^+^ cells is noted at any time point investigated compared to untreated controls. Furthermore, densities of Olig2^+^ cells are significantly higher on day 21 after the first injection and on day 3 after the fourth injection in rMOG-immunized mice. Data are expressed as mean + SEM. For statistical evaluation, one-way ANOVA followed by post hoc LSD test is performed (**p < 0.01). *Scale bar* 25 μm

We then set out to examine cortical Olig2^+^ OPCs (Fig. 8a, b). Three days after the first lesion induction, the density of Olig2^+^ cells was not reduced in demyelinated cortex (45.8 ± 2.9 cells/mm^2^) and similar to non-lesioned controls (45.7 ± 2.5 cells/mm^2^, Fig. 8b). Three weeks after the first lesion induction, however, the density of Olig2^+^ cells was significantly increased in rMOG-immunized rats (60.1 ± 6.2 cells/mm^2^) compared to untreated controls (*p* < 0.01), to rMOG-immunized rats on day 3 (*p* < 0.01) and to cytokine-injected controls (40.8 ± 1.0 cells/mm^2^; *p* < 0.01).

To examine OPC proliferation after lesion induction, Olig2 and BrdU double-positive cells were quantified in the cortex in animals pretreated with the proliferation marker BrdU. Three weeks after single lesion induction, the density of Olig2/BrdU double-positive cells was significantly increased in previously demyelinated cortex as compared to the non-demyelinated control group (Suppl. Fig. 2).

Following the second and fourth demyelinating episode, the Olig2^+^ cell density was already increased on day 3 after the last injection in rMOG-immunized rats (2nd injection 54.1 ± 3.1 cells/mm^2^, *p* < 0.05; 4th injection 59.9 ± 5.2 cells/mm^2^) compared to repeatedly injected controls (Fig. 8a, b). Furthermore, 3 weeks after the second and fourth injection, the numbers of Olig2^+^ cells were unaltered in rMOG-immunized rats compared to controls. These findings therefore indicate that OPC numbers were not reduced within SCD even after repeated lesion induction.

### Efficient remyelination after transient acute demyelination in rats with repeated focal lesioning

To compare cortical de- and remyelination after repeated lesioning, animals were evaluated on day 3 for demyelination and on day 21 for remyelination after final lesion induction following one, two and four stereotactic cytokine injections.

**Fig. 9:**
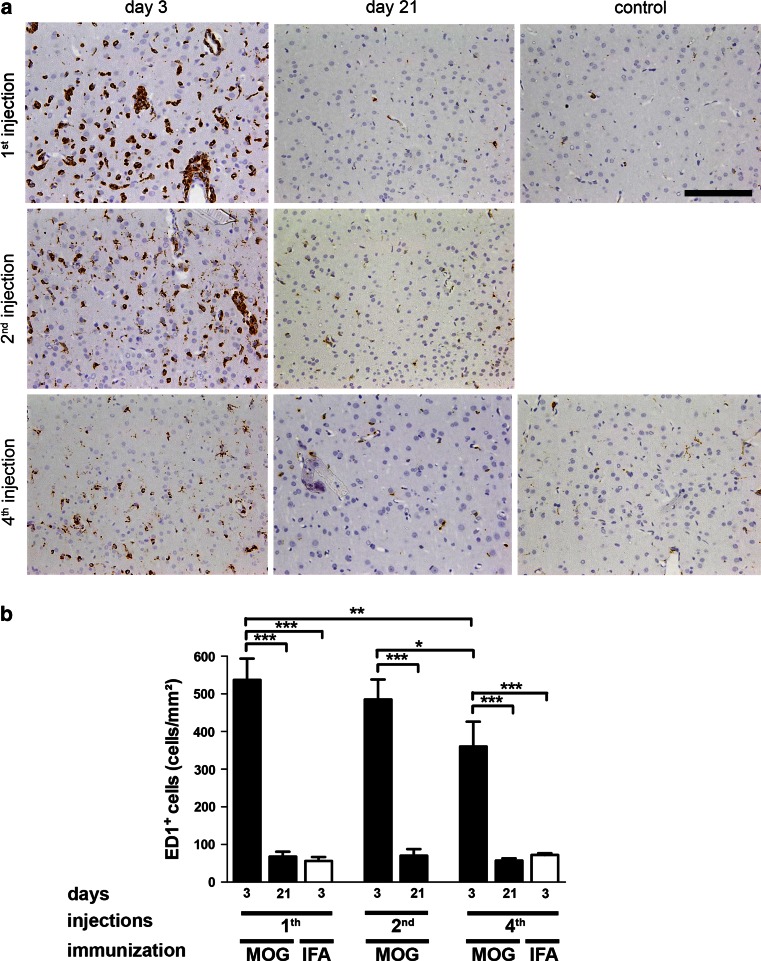
Transient increase of activated macrophages and microglia shortly after single and repeated cytokine injection in rats. **a** Representative photographs show ED1-immunostained sections within the cytokine-injected cortex on days 3 and 21 after last lesion induction of the indicated experimental groups. **b** Quantification of ED1^+^ activated macrophages and microglia within cortical layer III. On day 3 after lesioning, the density of ED1^+^ macrophages and microglia is significantly higher in rMOG- than IFA-immunized animals after single or repeated, respectively, cytokine injection(s). Data are expressed as mean + SEM. For statistical evaluation one-way ANOVA followed by post hoc LSD test is performed (*p < 0.05, **p < 0.01, ***p < 0.001). *Scale bar* 100 μm

First, we examined whether repeated cytokine injection was associated with the activation of microglia and macrophages in rMOG-immunized animals (Fig. 9). Three days after single, twofold and fourfold lesion induction, the density of activated ED1^+^ microglia and macrophages was substantially increased in layer III of the ipsilateral cortex (1st injection 537.1 ± 56.6 cells/mm^2^, 2nd injection 485.3 ± 52.8 cells/mm^2^, 4th injection 360 ± 66.3 cells/m^2^) in rMOG-immunized rats compared to cytokine-injected control animals (1st injection 56.0 ± 10.7 cells/mm^2^, 4th injection 72 ± 4.6 cells/m^2^, *p* < 0.001). The infiltration with ED1^+^ cells was transient in rMOG-immunized animals and dropped again to control levels on day 21 after last lesion induction (Fig. 9).

**Fig. 10:**
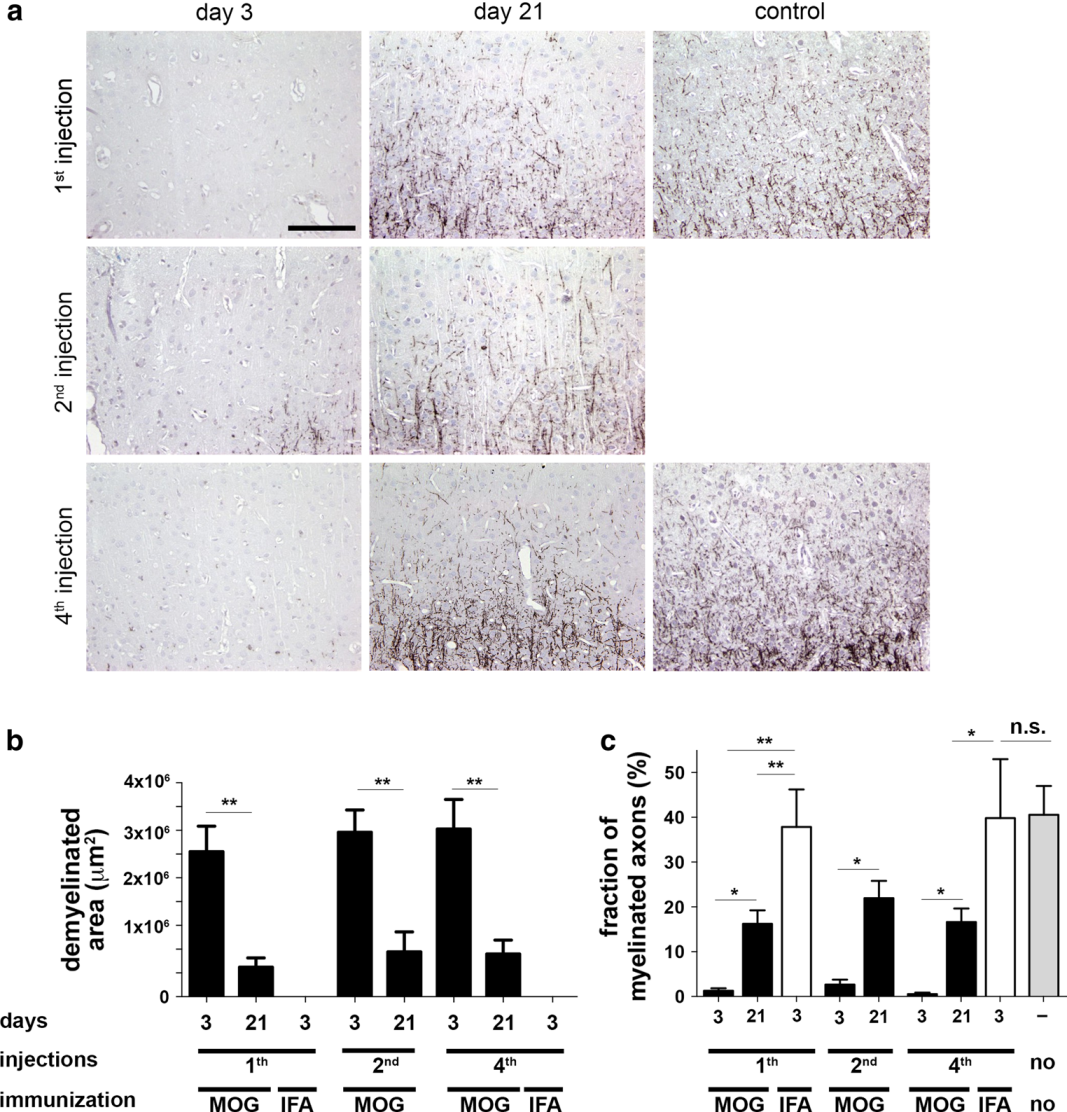
Unaltered remyelination after repeated cortical demyelination in rats. **a** Representative photographs show MBP-immunostained sections within cytokine-injected cortex on days 3 and 21 after last lesion induction in rMOG-immunized animals and controls. **b** Quantification of the demyelinated cortical area in rMOG- or IFA-immunized animals following intracerebral cytokine injection. Animals immunized with rMOG show a similar size of cortical demyelination on day 3 after single or repeated lesion induction. Likewise, the area of cortical demyelination is consistently decreased in these experimental groups on day 21 after single or repeated lesioning. In contrast, IFA-immunized controls (“IFA”) show no signs of demyelination. **c** Quantification of the myelinated axon fraction reveals a significant reduction of the myelinated axon fraction in demyelinated (day 3) animals compared to remyelinated (day 21) animals and controls. The fraction of myelinated axons remains stable at the remyelination time point (day 21) after single versus repeated injection, but is still significantly lower than in controls. Data are expressed as mean + SEM. For statistical evaluation, one-way ANOVA followed by post hoc LSD test is performed (**p < 0.01, ***p < 0.001). *Scale bar* 100 μm

SCD appeared widespread on MBP-immunostained sections following 3 days after single, two- or fourfold cytokine injection in rMOG-immunized rats (Fig. 10a). In contrast, IFA-immunized controls did not show any signs of demyelination on day 3 after the final cytokine injection (Fig. 10a). In rMOG-immunized animals on day 3 following the last injection, there was a similar degree of cortical demyelination in rats after one (1.5 ± 2.7 mm^2^), two (1.7 ± 0.2 mm^2^) or four (1.8 ± 0.3 mm^2^) demyelinating episodes (Fig. 10b). All rMOG-immunized animals showed a significant reduction of the demyelinated area at 3 weeks after final lesion induction compared to the corresponding group on day 3 (1st injection 0.3 ± 0.1 mm^2^, *p* < 0.001, 2nd injection 0.5 ± 0.2 mm^2^, *p* < 0.01, 4th injection 0.4 ± 0.1 mm^2^, *p* < 0.001). The size of the demyelinated cortical area after 3 weeks was similar in all rMOG-immunized animals—irrespective of the number of cytokine injections (Fig. 10b). These findings indicate that cortical remyelination is not impaired even after repeated inflammatory demyelination within the same area.

To assess the proportion of remyelinated axons, we quantified axons and myelin fibers after repeated lesioning. Axonal morphology appeared normal, adjacent to the injection site (Suppl. Fig. 3), and axonal densities in layer III resembled untreated control levels after one, two and four stereotactic cytokine injections (Suppl. Fig. 3). To examine whether the density of remyelinated fibers remained stable following recurrent cortical demyelination, we calculated the ratio between the density of myelinated fibers and axons in cortical layer III at different time points (Fig. 10c). In controls the fraction of myelinated cortical axons was 40.5 ± 6.4 %. At the demyelination time point (day 3 after the last injection) the proportion of myelinated axons was markedly decreased (1st injection 1.3 ± 0.6 %, 2nd injection 2.7 ± 1.1 %, 4th injection 0.5 ± 0.3 %) in rMOG-immunized rats. Three weeks after the last lesion induction, rMOG-immunized rats displayed a significantly higher proportion of myelinated axons than at the previous demyelination time point (*p* < 0.01 for 1st, 2nd and 4th injection). Furthermore, a similar fraction of myelinated axons was detected at the remyelination time point after one (16.2 ± 3.0 %), two (22.0 ± 3.8 %) and four (16.6 ± 3.0 %) stereotactic injections (Fig. 10c). Taken together, these data suggest that the rat cerebral cortex shows a high degree of endogenous oligodendroglial regeneration and remyelination capacity which does not seem to diminish even after four cycles of de- and remyelination in the targeted EAE model.

## Discussion

Cortical demyelinated lesions in chronic MS patients were recently shown to be widespread and even to exceed WM lesions with regard to lesion volume [19, 30]. Here, we investigated whether the densities of OLs and OPCs were altered in cortical lesions of early and late-stage MS. Furthermore, we examined the efficacy of OPC and OL recruitment in cortical demyelinated lesions in a rat model of MS. Our data indicate that NogoA^+^ OLs as well as Olig2^+^ OPCs are substantially reduced in chronic, but not early cortical MS lesions. In our animal model, NogoA^+^ cell density was only transiently reduced in the affected cerebral cortex, but rapidly recovered to control levels even after four demyelinating episodes.

Remyelination is considered to be an important mechanism for long-term axonal protection and is thus of enormous therapeutic interest. Accordingly, numerous studies have investigated the factors promoting remyelination in WM MS lesions [15]. Interestingly, the vast majority of early WM lesions harbor abundant NogoA^+^ and Olig2^+^ oligodendroglia and even contain newly formed myelin sheaths that enwrap previously demyelinated axons [33]. Although there is evidence for a variable degree of remyelination in around 40 % of chronic WM lesions, complete remyelination is rare [21]. Furthermore, chronic late-stage WM lesions contain hardly any mature OLs and also only few OPCs. Cortical lesions show evidence for more extensive remyelination at the lesion border compared to chronic WM lesions [2, 12]. Nevertheless, persistent cortical demyelination is an important feature of progressive MS, indicating that cortical remyelination fails at a certain disease stage. It remains unclear which factors contribute to OL demise and the failure of oligodendroglial differentiation in chronic cortical MS lesions. Our findings of relatively preserved axonal densities within chronic SCD argue against marked axonal loss as the decisive factor for cortical remyelination failure. Even though we cannot rule out the possibility that subtle axonal changes and degeneration may still occur in MS GM, such alterations are unlikely to result in OPC loss and the complete lack of remyelination observed in chronic SCD. It seems likely that other factors such as loss of local signals required for OPC survival and migration [39, 49, 51] contribute to remyelination failure. Furthermore, microglial, astrocytic and neuronal changes might contribute to reduced myelin regeneration in MS cortex.

One hypothesis investigated in the present study was that multiple demyelinating events may lead to failure of remyelination. This is mainly supported by experimental models of the disease [32]. Repeated or sustained episodes of demyelination might finally exhaust the ability of OPCs to divide and differentiate into myelinating OLs. Alternatively, OPCs might be depleted from repeatedly demyelinated lesions and be unable to repopulate lesions from the periphery. In addition, multiple demyelinating episodes might furthermore change the tissue microenvironment and the susceptibility of axons to myelination.

In the present study we investigated if multiple cortical demyelinating events lead to the reduction of mature OLs and OPCs, thus limiting the extent of cortical remyelination. We employed a targeted EAE model based on subclinical immunization with rMOG [38]. This model reproduces SCD found in MS patients and allows for precise lesion localization and timing. Our data indicate that repeated induction of cortical demyelination does not substantially affect the recruitment of OPCs, the generation of mature OLs or remyelination in rats. It should be noted, however, that a temporary reduction in mature OLs was observed after four cytokine injections, but OL density recovered at later time points, indicating a slight delay in recruitment.

Our findings are in line with previous experimental work that relied on non-immunological mechanisms of demyelination. Efficient remyelination was observed after three repeated injections of ethidium bromide into the cerebellar peduncle [40]. Similarly, unaltered remyelination capacity was also observed in mice after two episodes of toxic cuprizone-induced demyelination [25]. These results in rodent models of CNS demyelination with good preservation of axons, driven by inflammation or by gliotoxic substances, highlight that endogenous remyelination capacity is not substantially impaired even after repeated episodes of demyelination.

In contrast to these experimental findings, patients with long-standing MS showed oligodendroglial depletion in chronic SCD as evidenced by reduced NogoA^+^ OLs and Olig2^+^ OPCs. The reduction of OPCs in chronic SCD suggests that failure of cortical remyelination cannot simply be attributed to differentiation failure of OPCs, as it was suggested for WM MS lesions [11, 29, 42]. Furthermore, the strong reduction in OLs and OPCs observed in long-standing human cortical MS lesions cannot be mimicked by recurrent demyelinating events in our experimental model. One might speculate that four courses of demyelination are not sufficient to exhaust the remyelinating capacity in the rat cortex. It is also conceivable that our model, which is based on anti-MOG antibody-mediated myelin destruction, may not fully mimic the pathogenesis of MS, where the target(s) of the immune reaction and the contribution of anti-myelin antibodies have not yet been determined. In addition, it is well known that remyelination in the rodent brain is a very robust phenomenon. As such, the loss of OLs and OPCs seen in chronic MS may represent a phenomenon that is specific to the disease process in humans. Our model thus is suited to study early lesion formation, but does not lead to the late-stage oligodendroglial deficiency and inability to remyelinate as found in chronic MS lesions.

In early MS biopsies, NogoA^+^ mature OLs did not show any reduction in demyelinated cortical lesions, whereas a transient decrease was observed in the animal setting. However, it is important to note that the lesion stage of SCD is likely to differ between early human biopsy cases and rats with induced SCD. The animal model allows immediate investigations within 3 days after lesion induction, whereas this is not possible in patients who had already undergone a set of diagnostic investigations prior to biopsy. Given these temporal differences, it may not be surprising that proliferating OPCs were only observed in rat, but not in human early SCD. Whether mature OLs observed in early MS lesions represent preserved or already repopulated OLs cannot be distinguished based on these single time point biopsies. Interestingly, we noted a trend toward higher OPC numbers in cortical layer I/II in MS biopsies which might indicate previous OPC proliferation.

One important factor associated with altered remyelination capacity and disease progression is aging [14]. In the current study we used up to 4- to 5-month-old rats with a follow-up period of up to 4 months. In contrast, human MS autopsy cases in this study had an average age of 54 years and showed a progressive disease course for at least 10 years. Therefore, experiments in older animals might be required to test whether repeated induction of SCD would finally result in impaired cortical remyelination. Previous experimental studies showed reduced recruitment of OPCs into the lesion area and impaired oligodendroglial differentiation in older animals [46, 47]. Furthermore, increased axonal vulnerability and decreased remyelination efficiency were recently reported in aged rats after targeted demyelination in the spinal WM [22]. Similarly, remyelination is less efficient in aged animals after lysolecithin [20] or cuprizone [45] induced demyelination. Mechanisms contributing to such age-related effects may involve intrinsic determinants such as age-dependent epigenetic control of gene expression of OPCs [45] and extrinsic factors acting on OPCs in the lesion microenvironment [23, 52].

A recent study investigated the presence of NG2^+^ polydendrocytes in cortical demyelinated MS lesions and found similar densities in cortical demyelinated lesions, normal-appearing and control cortex [12]. Genetic fate mapping indicates that NG2^+^ polydendrocytes can generate both OLs and astrocytes in GM [53]. Interestingly, constitutive deletion of Olig2 in NG2^+^ cells leads to their conversion into astrocytes in the neocortex and corpus callosum [54]. These findings suggest that Olig2 is crucial for the differentiation of NG2^+^ polydendrocytes into OLs in the cerebral cortex. Thus, NG2^+^ polydendrocytes lacking Olig2 expression in chronic SCD may have lost their capacity to differentiate into myelin-forming OLs.

The precise pathogenetic mechanisms leading to SCD are still not known. However, chronic T- and B-lymphocytic inflammation in the meninges was associated with shorter time to progression and more severe clinical disease in secondary progressive MS [36]. Meningeal inflammation and cortical demyelination were also recently identified as important features of primary progressive MS, which strongly suggests that inflammation and “neurodegeneration” are closely linked phenomena in MS [13]. Recent experimental findings indicate that the subarachnoid injection of proinflammatory cytokines is sufficient to induce cortical demyelination in MOG-immunized animals [16]. Consequently, chronically elevated cytokine levels may impede the proliferation, differentiation and survival of cortical OLs, and finally lead to persistent demyelination.

In conclusion, we show that oligodendroglial cells are present in early cortical MS lesions, but substantially decreased in chronic SCD. The latter could not be modeled in our experimental studies in which recruitment of OLs and remyelination were not impaired even after repeated cortical demyelination. This suggests that failure of cortical remyelination in chronic MS may not simply result from repeated episodes of cortical demyelination affecting mature OLs, but may also require other factors damaging OPCs at an early differentiation stage. Further studies are needed to unravel the factors involved and mechanisms responsible for the cortical remyelination failure observed in progressive MS.

## Electronic supplementary material

Below is the link to the electronic supplementary material.

**Suppl. Fig. 1. Preserved axonal density in chronic cortical MS lesions.** (**a**) Axonal densities are assessed in layer III on Bielschowsky silver-stained sections. Representative images of Bielschowsky silver-stained sections of layer III in non-MS neocortex (control), normal-appearing MS cortex (NAGM) and chronic SCD. (**b**) Quantification of axonal densities in layer III in control cortex, NAGM and chronic SCD. Data are expressed as mean + SEM. Scale bar: 20 μm. (TIFF 4734 kb)

**Suppl. Fig. 2. OPC proliferation after remyelination in rats. (a)** The proliferated OPC population is determined in SCD by Olig2 (red) and BrdU (green) double immunofluorescence. (**b**) The density of Olig2/BrdU double-positive cells is significantly increased in previously demyelinated cortex at 21 days post lesion induction compared to controls (s.c. IFA). Data are expressed as mean + SEM. *** = p < 0.001. (TIFF 1243 kb)

**Suppl. Fig. 3. Preserved axonal density after repetitive demyelinating episodes in rats. (a)** Representative photographs of Bielschowsky silver-stained sections of layer III of cortical lesions 21 days after cytokine injection. The different immunization protocols (s.c. rMOG or s.c. IFA, respectively) are arranged in columns. The numbers of lesion inductions (1st, 2nd and 4th) are arranged in rows. (**b**) Axonal densities are similar in rMOG- and IFA-immunized rats within the center of the lesion in cortical layer III after single or repeated injection(s). Data are expressed as mean + SEM. Scale bar: 20 μm. (TIFF 3424 kb)
