# Supplementary figures and images for: Oligodendroglia in cortical multiple sclerosis lesions decrease with disease progression, but regenerate after repeated experimental demyelination
Source: Acta Neuropathol. 2014 Feb 25;128(2):231–46. doi: 10.1007/s00401-014-1260-8 (PMC4102825; doi:10.1007/s00401-014-1260-8)

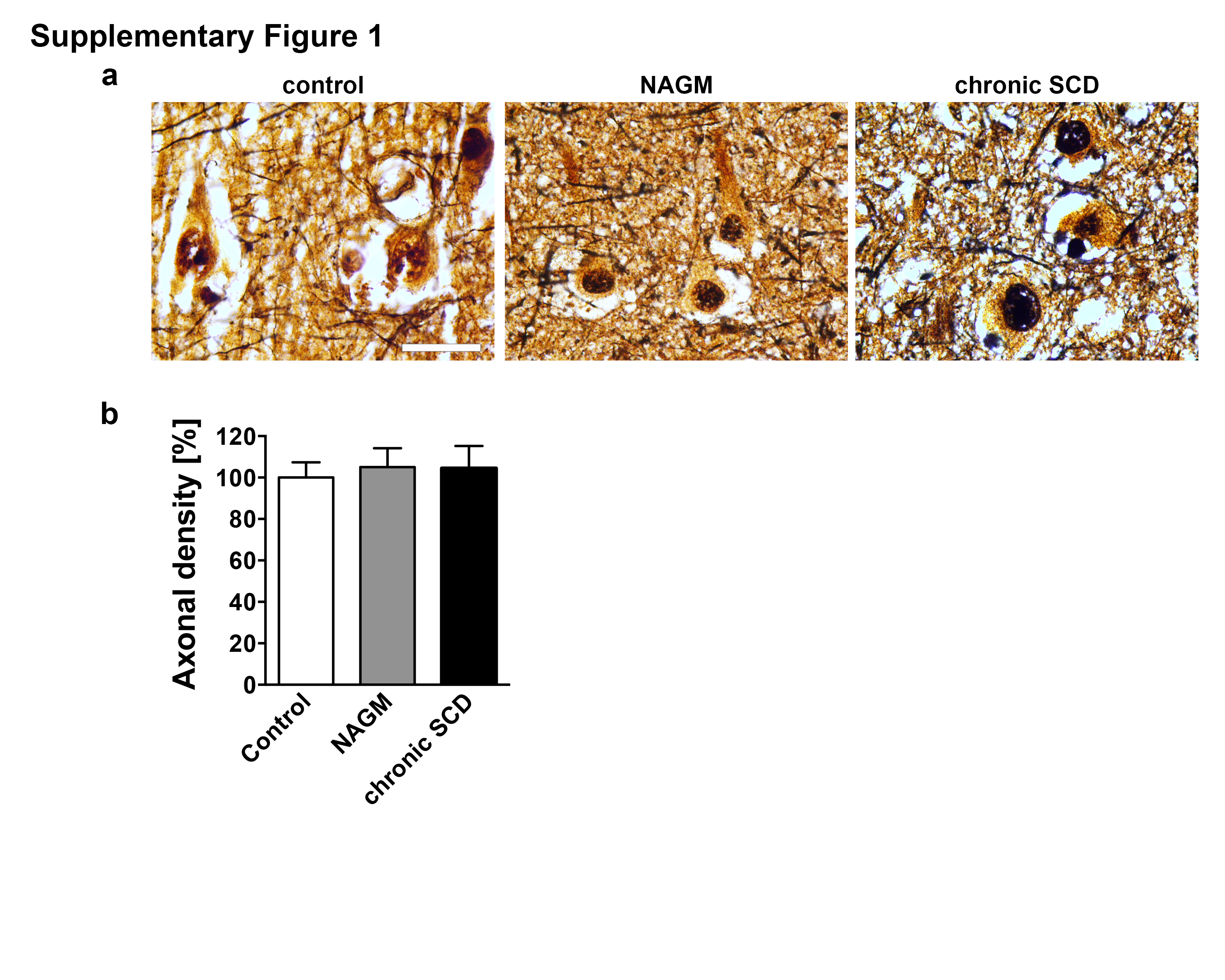

Supplement: Supplementary file 1 — Suppl. Fig. 1. Preserved axonal density in chronic cortical MS lesions. (a) Axonal densities are assessed in layer III on Bielschowsky silver-stained sections. Representative images of Bielschowsky silver-stained sections of layer III in non-MS neocortex (control), normal-appearing MS cortex (NAGM) and chronic SCD. (b) Quantification of axonal densities in layer III in control cortex, NAGM and chronic SCD. Data are expressed as mean + SEM. Scale bar: 20 μm. (TIFF 4734 kb) [file 401_2014_1260_MOESM1_ESM.tif]

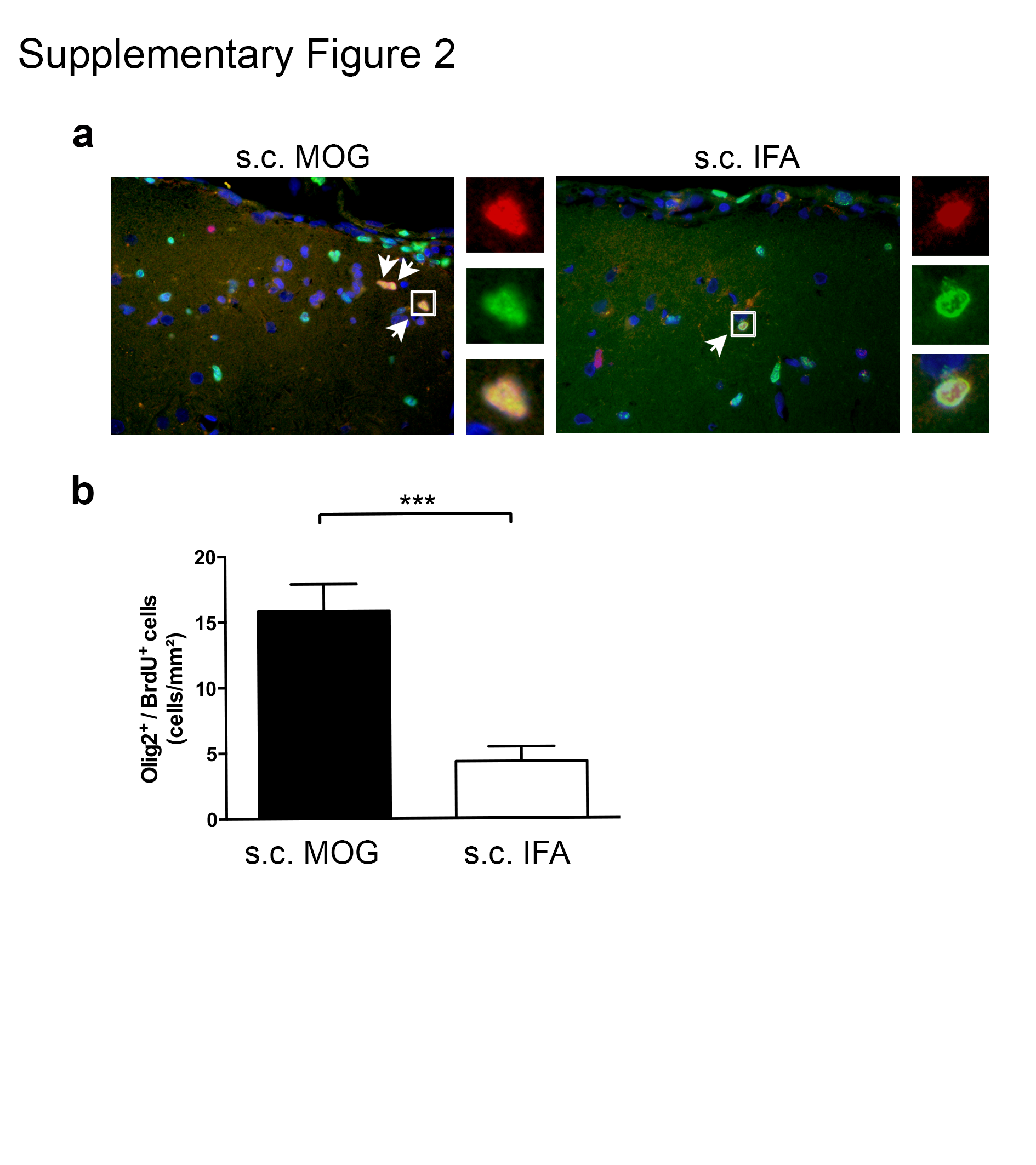

Supplement: Supplementary file 2 — Suppl. Fig. 2. OPC proliferation after remyelination in rats. (a) The proliferated OPC population is determined in SCD by Olig2 (red) and BrdU (green) double immunofluorescence. (b) The density of Olig2/BrdU double-positive cells is significantly increased in previously demyelinated cortex at 21 days post lesion induction compared to controls (s.c. IFA). Data are expressed as mean + SEM. *** = p < 0.001. (TIFF 1243 kb) [file 401_2014_1260_MOESM2_ESM.tif]

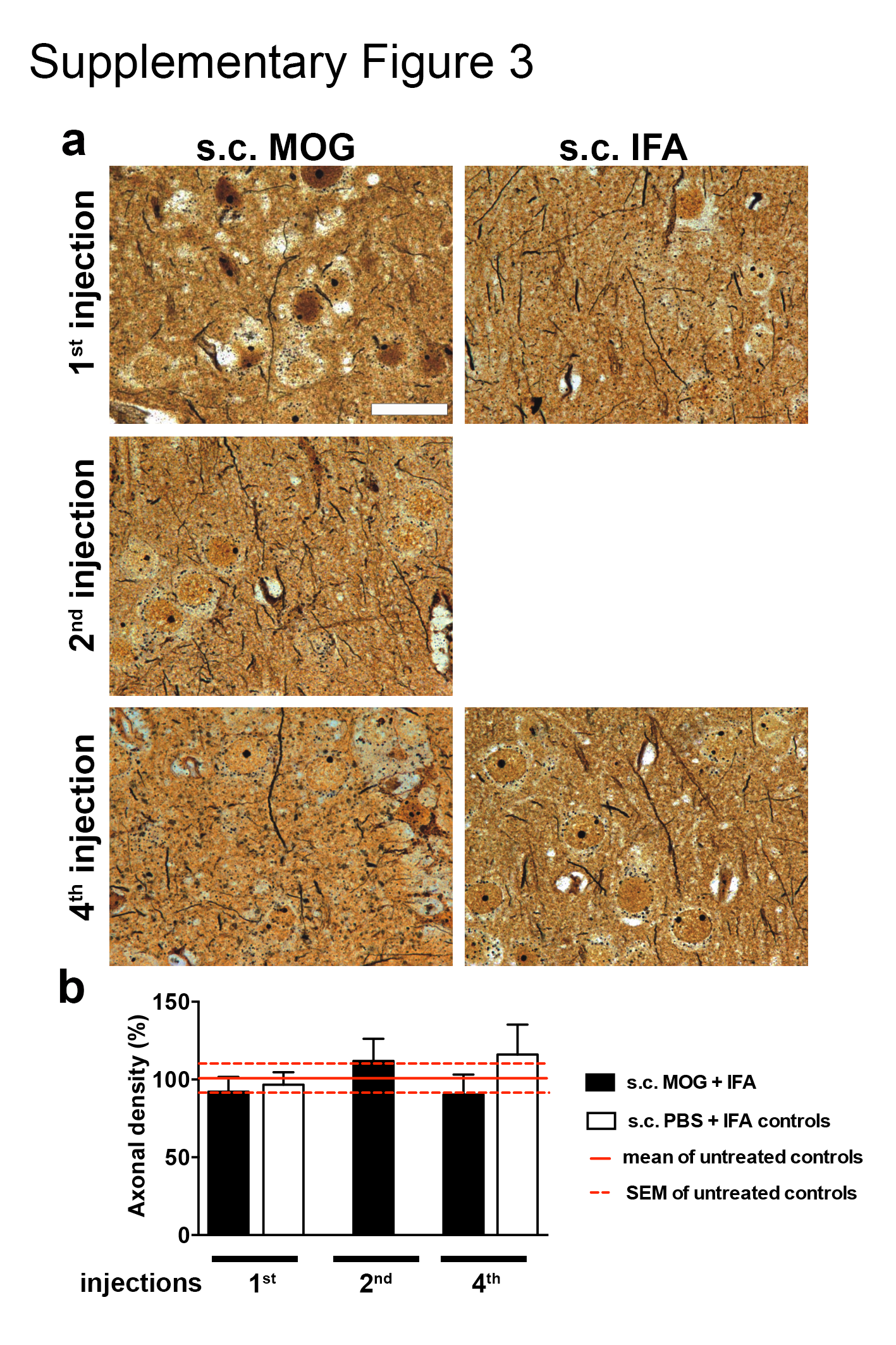

Supplement: Supplementary file 3 — Suppl. Fig. 3. Preserved axonal density after repetitive demyelinating episodes in rats. (a) Representative photographs of Bielschowsky silver-stained sections of layer III of cortical lesions 21 days after cytokine injection. The different immunization protocols (s.c. rMOG or s.c. IFA, respectively) are arranged in columns. The numbers of lesion inductions (1st, 2nd and 4th) are arranged in rows. (b) Axonal densities are similar in rMOG- and IFA-immunized rats within the center of the lesion in cortical layer III after single or repeated injection(s). Data are expressed as mean + SEM. Scale bar: 20 μm. (TIFF 3424 kb) [file 401_2014_1260_MOESM3_ESM.tif]
